# Delayed intra-abdominal bleeding following trans-vaginal ultrasonography guided oocyte retrieval for *in vitro* fertilization in patients at risk for thrombo-embolic events under anticoagulant therapy

**DOI:** 10.12688/f1000research.2-189.v2

**Published:** 2013-10-18

**Authors:** Roy Mashiach, David Stockheim, Mati Zolti, Raoul Orvieto

**Affiliations:** 1 Department of Obstetrics and Gynecology, Chaim Sheba Medical Center, Ramat Gan, Israel; 2 Sackler Faculty of Medicine, Tel Aviv University, Tel Aviv, Israel; 3 Faculty of Health Sciences, Ben Gurion University of the Negev, Beer Sheva, Israel

## Abstract

We report herein, two cases of massive delayed (2 and 4 days) intra abdominal hemorrhage following ovum pick-up (OPU), in patients at risk for thrombo-embolic events, who concomitantly used therapeutic doses of low molecular weight heparin (LMWH). We discuss the possible mechanisms involved in causing the aforementioned delayed bleeding, and call for re-evaluation of the presently accepted anticoagulant co-treatment regimen. These case reports should direct physicians' attention and keep them alert, while conducting IVF treatment to this subgroup of high risk patients.

## Introduction

Nowadays, ultrasonographically guided trans-vaginal ovum pick-up (OPU) is considered an accepted and safe method for oocyte retrieval worldwide. Nevertheless, the multiple punctures of the vaginal vault can injure or tear the vaginal mucosa, ovaries, intra-abdominal organs or blood vessels, resulting in mild to severe internal and external bleeding and other complications ^1, 2^. Clinically significant blood loss after OPU is actually uncommon ^3^, with a reported incidence of severe intra- or retroperitoneal bleeding varying from 0 to 1.3%; ^2^.

Intraperitoneal bleeding tends to be severe with acute hemodynamic deterioration, whereas retroperitoneal bleeding usually has a later and more indolent presentation. Intra-abdominal bleeding should be suspected immediately after OPU with the development of signs and symptoms of anemia. While a drop in hemoglobin concentration is an indication for prompt blood transfusion, hemodynamic deterioration dictates surgical intervention with subsequent hemostatis. Most of cases are diagnosed several hours following OPU, with a reported interval between OPU and surgical intervention, ranging from 3 to 18 hours ^4, 5^.

In patients with an underlying coagulopathy, the interval between OPU and the appearance of clinical signs and symptoms of severe intra-abdominal bleeding or surgical intervention, was reported to be longer. While, Battaglia *et al.* ^6^ reported a 3 hour interval between OPU and surgical intervention in a patient with coagulation factor XI deficiency ^6^, intervals of 2 and 10 days, were reported in patients suffering from essential thrombocythemia ^7^ and factor VIII deficiency ^8^, respectively. Notably, none of the aforementioned patients were treated with anticoagulant medications.

The situation is even more complicated for women with known thrombophilia and/or history of a thrombo-embolic event, who use anticoagulant drugs. The hitherto published research regarding this issue is scarce, with only one study reporting on 24 patients treated concomitantly with anticoagulation therapy, who underwent 68 oocyte retrieval procedures ^9^. Since IVF treatment was not associated with any medical complication, such as bleeding or thromboembolic complications, Yinon *et al.* have concluded that among patients considered as high risk for a thromboembolic event, the introduction of low molecular weight heparin (LMWH), as a cycle protective treatment, is safe ^9^.

Here, we report two cases of massive delayed intra-abdominalhemorrhage following OPU, in patients at risk for thromboembolic events, who concomitantly used LMWH. These cases aim to direct physicians' attention and keep them alert, while treating this subgroup of at risk patients.

## Case report

Two patients were referred to our IVF clinic for egg/embryos collection for surrogacy because of the risk associated with pregnancy. Both were under therapeutic doses of LMWH, which according to a senior haematologist consultant, were co-administered during the controlled ovarian hyperstimulation (COH) for IVF, until 16 h before the oocyte retrieval and resumed 12 h after the procedure. In both cases, the follicles were aspirated by experienced physicians using a fine (19 G) needle (William A. Cook Australia PTY LTD, Australia) and 140 mmHg suction pressure. In each ovary, the follicles were sequentially punctured without reinserting the needle through the vaginal wall. This study was approved by the institutional review board of Sheba Medical Centre.

### Case 1:

A 37-year-old patient with essential thrombocytosis, was diagnosed incidentally following routine examination 7 years ago. One year ago, she developed sinus vein thrombosis, necessitating therapeutic doses of anticoagulant therapy. Controlled ovarian hyperstimulation included the multidose GnRH antagonist protocol, with follicle stimulating hormone (FSH) daily dose of 112.5 to 150 IU. She achieved a peak E2 level of 1538 pmol/l with one leading follicle at each ovary. OPU was reported as uneventful with a retrieval of 3 oocytes, of with 2 were fertilized and cryopreserved. A routine complete blood count (CBC) 2 h following OPU revealed a stable hemoglobin (Hb) concentration, as compared to her baseline level (9.92 g/dL vs 10.52 g/dL, respectively).

Two days following OPU she was re-admitted to our ward, because of severe abdominal and shoulder pain, abdominal bloating and tenesmus. On physical examination her abdomen was swollen and tender, with floating blood as evidenced by transabdominal ultrasonography. She was hospitalized and closely observed hemodynamically. During the next 4 h, her Hb concentration dropped to 7.2 g/dL with clinical deterioration, mandating resuscitation, and urgent exploration.

During exploratory laparoscopy, a massive hemoperitoneum was found (approximately 2.5 L of blood), and a profuse bleeding was observed from a large vein in the posterior aspect of the left infundibulo-pelvic ligament. The vessel was successfully coagulated. The patient was transfused with 4 units of blood, 3 unit of cryoprecipitate, and 2 units of fresh frozen plasma. Her postoperative course was uneventful and she was discharged 4 days later with a hemoglobin level of 9.4 g/dL.

### Case 2:

A 32-year-old patient, suffering from Budd–Chiari syndrome as a consequence of a myeloprolifertive disorder, necessitating therapeutic doses of anticoagulant therapy. Controlled ovarian hyperstimulation included the long GnRH agonist protocol, with HMG daily dose of 375 to 450 IU. She achieved a peak E2 level of 2349 pmol/l with four leading follicles at both ovaries. OPU was reported as technically uneventful, however no oocytes were retrieved (empty follicles).

Three days following OPU, the patient started to feel unwell and complained of lower abdominal pain and dyspnea with stable Hb concentration (10.43–10.95 g/dL). A day later, she gradually became pale, tachycardiac, with a drop in Hb level (8.84 g/dL) that continued (8.66 g/dL) despite a blood transfusion, mandating urgent exploratory laparoscopy. During laparoscopy, a massive hemoperitoneum was observed (approximately 2.5 L of blood), with an active bleeding from a tear of the right ovarian capsule. The tear was successfully coagulated with an accurate hemostasis. The patient was transfused with 5 units of blood, and 2 units of fresh frozen plasma. Her postoperative course was uneventful and she was discharged a week later with a hemoglobin level of 11.68 g/dL.

## Discussion

We report herein, to our knowledge, the first 2 cases of massive delayed intra-abdominal hemorrhage following OPU, in patients at risk for thromboembolic event, who concomitantly used *therapeutic* doses of LMWH. We are not aware of any guidelines that have been published by any professional society, regarding OPU under LMWH. Moreover, since most guidelines relate to patients undergoing surgeries that may apply strict hemostatic measures, which are not available during US guided OPU, our report challenges and calls for re-evaluation of the anticoagulation regimen, provided to this subgroup of at risk patients, during an IVF treatment cycle.

Following OPU, the cessation of bleeding requires the interaction of the injured blood vessel with the circulating platelets and clotting proteins in order to form a stable platelet-fibrin clot. Any abnormalities of these factors may result in clinically significant bleeding. In the present cases, both patients received therapeutic doses of LMWH (enoxaparin, 60mg bid), which were discontinued 16 h prior to OPU and resumed 12 h after the procedure. Moreover, intra abdominal bleeding occurred 2 and 4 days following OPU.

Several plausible mechanisms might be responsible for the aforementioned delayed bleeding: (a) early postoperative use of enoxaparin and unfractionated heparin was reported to be associated with a significant increase in re-exploration for postoperative bleeding, often at a significantly delayed time period after the initial surgery ^10^. Moreover, this delay was especially common with enoxaparin; (b) while the effect of LMWH and unfractionated heparin on the coagulation cascade is well established, we may wonder whether these substances also affect the fibrin nanostructure, which plays a major role in the mechanical strength and lysis of clots. Yeromonahos *et al.* ^11^ provided quantitative evidence showing that both LMWH and unfractionated heparin, independent of their other actions on the coagulation cascade, directly alter the nanostructure of fibrin fibers, contributing to improved fibrinolysis.

We therefore believe that the presently accepted anticoagulant co-treatment regimen, including the dose and timing of administration, should be challenged and undergo a critical re-evaluation. For example, delaying *(24 h after the procedure)* and reducing the first dose ( *half*) of LMWH post OPU, with gradual increments ( *after 2 days of half a daily dose*) might offer some advantages. However, whether this modification will increase the tendency toward thrombosis, should be further investigated by a *multidisciplinary experts*. Moreover, since intra abdominal bleeding obviously occurs, and may occur several days following OPU, we recommend that the patient be kept in the ward for observation for at least 2–4 days following OPU.

In the presented cases we also observed a tear of the ovarian capsule and an inadvertent puncture of a blood vessel within the infudibullo-pelvic ligament. Both complications might have been prevented by meticulous follicular aspiration using a small diameter aspiration needle (19G), and preferably under the guide of color Doppler ultrasonography ^12^, respectively.

In conclusion, massive delayed intra-abdominal hemorrhage may occur following OPU, in patients at risk for thromboembolic event, who concomitantly used LMWH. These case-reports should direct physicians' attention and keep them alert, while conducting an IVF treatment to this subgroup of high risk patients.
