# Supplementary material for: Delayed intra-abdominal bleeding following trans-vaginal ultrasonography guided oocyte retrieval for in vitro fertilization in patients at risk for thrombo-embolic events under anticoagulant therapy
Source: F1000Res. 2013 Oct 18;2:189. Originally published 2013 Sep 16. [Version 2] doi: 10.12688/f1000research.2-189.v2 (PMC3869527; doi:10.12688/f1000research.2-189.v2)
Supplement: Peer review history

# Peer review: Referee response for version 2

**Published:** 2013-11-27

**Authors:** Pedro Barri

**Affiliations:** 1 Department of Obstetrics, Gynaecology and Reproduction, Institut Universitari Dexeus of the Universitat Autònoma de Barcelona, Barcelona, Spain

I have read this submission. I believe that I have an appropriate level of expertise to confirm that it is of an acceptable scientific standard.

# Peer review: Referee response for version 2

**Published:** 2013-10-21

**Authors:** Fatma Ferda Verit

**Affiliations:** 1 Department of Obstetrics and Gynecology, Suleymaniye Maternity, Research and Training Hospital, Istanbul, Turkey

I accept this article as it stands.

I have read this submission. I believe that I have an appropriate level of expertise to confirm that it is of an acceptable scientific standard.

# Peer review: Referee response for version 1

**Published:** 2013-10-30

**Authors:** William Ledger

**Affiliations:** 1 Obstetrics and Gynaecology, University of New South Wales, Sydney, Australia

This article is built around 2 case reports of clinically significant intra-abdominal bleeding in the days following egg collection for the purposes of IVF, in patients receiving therapeutic doses of Heparin. The authors use the case reports as a focus for discussion of the management of patients prescribed this medication prior to IVF.

The reports are provisionally novel to warrant publication. The use of English is somewhat idiosyncratic and requires minor review. The text preceding the first case report includes the suggestion that both eggs and embryos may be collected for surrogacy. Only eggs can be collected, and this should be amended.

The most important part of the article is in the discussion. The authors suggest a review of anticoagulant co-treatment in the light of their cases. I do not think that their experience would be sufficient risk of thrombotic complications. This problem is mentioned but should be further highlighted by the authors. I would also suggest they further emphasize the use of a smaller gage aspiration needle for egg collection in these higher risk cases.

I have read this submission. I believe that I have an appropriate level of expertise to confirm that it is of an acceptable scientific standard.

# Response

**Published:** 2013-11-03

**Authors:** Raoul Orvieto

**Affiliations:** Director, Infertility and IVF Unit, Sheba Medical Center, Israel

We would like to thank Prof. Ledger for his valuable comments.

Eggs were collected and fertilized, and the embryos were frozen for future FET cycles to the surrogate. The issue of the thrombotic risk and the suggestion of a smaller gage needle both appear in the 2nd version.

# Peer review: Referee response for version 1

**Published:** 2013-10-09

**Authors:** Pedro Barri

**Affiliations:** 1 Department of Obstetrics, Gynaecology and Reproduction, Institut Universitari Dexeus of the Universitat Autònoma de Barcelona, Barcelona, Spain

This is an observational case-report on two cases of delayed intra-abdominal bleeding following OPU for IVF in patients under anticoagulant therapy.

This article is acceptable, but some changes should be included:

 

1. There is no information either on the type of needles used for aspiration, or on the aspiration pressure.
   
2. Why does a young patient (32 yrs) have a negative OPU?
   
3. How many needle punctures have been performed?
   
4. There is no information on the outcomes of the oocytes retrieved in case 1.
   
5. The discussion section is well structured, but authors should present a final recommendation on daily doses and the duration of LMWH.

I have read this submission. I believe that I have an appropriate level of expertise to confirm that it is of an acceptable scientific standard.

# Response

**Published:** 2013-10-10

**Authors:** Raoul Orvieto

**Affiliations:** Director, Infertility and IVF Unit, Sheba Medical Center, Israel

We would like to thank Prof. Barri for his valuable comments.

1. The follicles were aspirated by experienced physicians using a fine (19 G) needle (William A. Cook Australia PTY LTD, Australia) and 140 mmHg suction pressure.

2. The negative OPU was unexplained (empty follicle?).

3. Punctures were started in the ovary with the follicles closest to the vagina, and each follicle was sequentially punctured without reinserting the needle through the vaginal wall.

4. In case 1, OPU was reported as uneventful with a retrieval of 3 oocytes, of which 2 were fertilized and cryopreserved.

5. As we already mentioned and further clarify: "We therefore believe that the presently accepted anticoagulant co-treatment regimen, including the dose and timing of administration, should be challenged and undergo a critical re-evaluation. For example, delaying *[24 h after the procedure]* and reducing the first dose [ *half*] of LMWH post OPU, with gradual increments *[after 2 days of half a daily dose*] might offer some advantages. However, whether this modification will increase the tendency toward thrombosis, should be further investigated [ *by multidisciplinary experts*]. Moreover, since intra abdominal bleeding obviously occurs, and may occur several days following OPU, we recommend that the patient be kept in the ward for observation for at least 2–4 days following OPU."

# Peer review: Referee response for version 1

**Published:** 2013-10-02

**Authors:** Fatma Ferda Verit

**Affiliations:** 1 Department of Obstetrics and Gynecology, Suleymaniye Maternity, Research and Training Hospital, Istanbul, Turkey

I think this article can be accepted without any revisions. The paper quality is good and it adds new knowledge to the literature.

I have read this submission. I believe that I have an appropriate level of expertise to confirm that it is of an acceptable scientific standard.
